# Dramatic Perezhivanie as a driver of executive functions development through role-play in early childhood: Theoretical framework and experimental evidence

**DOI:** 10.3389/fpsyg.2022.1057209

**Published:** 2022-11-18

**Authors:** Vera L. Sukhikh, Nikolay N. Veresov, Nikolay E. Veraksa

**Affiliations:** ^1^Department of Educational Psychology and Pedagogy, Lomonosov State Moscow University, Moscow, Russia; ^2^Psychological Institute of the Russian Academy of Education, Moscow, Russia; ^3^Faculty of Education, Monash University, Melbourne, SA, Australia

**Keywords:** role-play, imaginary situation, dramatic perezhivanie, social situation of development, early childhood, executive functions

## Abstract

Role-play in early childhood is associated with development of executive functions (EFs), although study results remain inconsistent. Due to the complex nature of the role-play, the underlying mechanisms of these associations are not obvious. In this article, play is viewed in the framework of the cultural-historical approach as a special social situation that can become the social situation of development if it results in dramatic perezhivanie of a child. In this study, we compared the level of EFs and play behavior between two play contexts: play guided by an adult and one with less adult involvement. Play behavior was analyzed based on five behavioral measures suggested to be the markers of dramatic perezhivanie. Measures of EFs were taken before and after the experimental procedure. Results show that dramatic perezhivanie might be considered a driver of EFs development through role-play in early childhood. As well as this, the involvement of an adult into play was associated with different patterns of EFs dynamics before and after the intervention. Future work can investigate if the construct of dramatic perezhivanie, microsocial situation of development, and micro-crisis might explain variability of the study results on the association between the role-play and child development.

## Introduction

The influence of play on the development of the executive functions (EFs) in preschool children has been widely studied (Blair and Diamond, 2008; McClelland et al., 2010; Diamond and Lee, 2011; Lillard et al., 2013; Goldstein and Lerner, 2018; Bukhalenkova et al., 2020; Thibodeau-Nielsen et al., 2020). A significant number of studies investigates how adults and child-adult interactions in play settings might influence the development of executive functions and support the idea that the self-regulation in early development is deeply embedded in the child’s relations with others (Hakkarainen et al., 2013; Fleer et al., 2020; Van Oers and Pompert, 2021; Veraksa and Veraksa, 2021; Veresov et al., 2021). At the same time, interesting new play-focused curricula emerged, for example, the Tools of the Mind (Bodrova and Leong, 2001, 2017) and playworlds (Lindqvist, 1996), both originated in Vygotsky’s theory of development. Tools of the Mind is a curriculum that started in 1993, but only in 2013/2014 has research been published to suggest that the Tools of the Mind Kindergarten program had a positive effect on executive functions, reasoning ability, and the control of attention (Bodrova et al., 2013; Blair and Raver, 2014). Similarly, the “playworlds” approach was developed in late 1990s and only recently has the experimental data appeared confirming its positive role in supporting children’s executive functions and self-regulation (Fleer, 2020; Walker et al., 2020). The researchers report that “playworlds are mostly theorized as collectively created imaginary situations with roles, rules and tasks, but in relation to the opportunities for the development of EF in children, the role of the adult seems to be a vital one and this needs further research” (Walker et al., 2020, p. 137). We believe our study might provide a research-based response to these challenges. However, there are methodological difficulties (i.e., role-playing is difficult to reproduce in the laboratory settings) and theoretical challenges (role-play and its structure are understood differently depending on the theoretical approach). It is also possible that play itself may be a product of other factors, which actually influence development (that is, the play becomes an epiphenomenon), or represents only one of the developmental pathways (equifinality; Lillard et al., 2013).

What needs to be done is to study the individual trajectories of development and identify indicators that show how play intervention influences EFs of a particular child. For now, there is no instrumental basis that allows the study of individual trajectories of development of EFs in young children. And finally, what needs further research is how different types of adult engagement in a role-play (play interventions) influence the development of EFs in children. We consider the cultural-historical approach introduced by Vygotsky (1998) and further developed in works of Luria (Luria, 1980; Vygotsky and Luria, 1993), Leontiev (1981), and Gal’perin (1969) to be fruitful in meeting these challenges. The theoretical framework of the article follows Vygotsky’s (2016) conception of play as an imaginary situation which by its duality provides conditions for cognitive development. Concepts of “dramatic perezhivanie” and “microsocial situation of development” are presented as theoretical analytical tools. In this paper, we theorize role-play as a social situation of a special kind, which potentially includes a series of microsocial situations of development. This theoretical framework allows for empirical examination of whether dramatic perezhivanie is a factor mediating the effect of play intervention on child’s development by transforming an imaginary situation from microsocial situation into microsocial situation of development.

### Role-play and imaginary situation

Vygotsky focused mainly on the sociodramatic role-play as it represents the peak of the development of the play in early childhood (Vygotsky, 2016). In this regard, the role-play is a more mature type of play compared to the director’s play (the child only constructs the plot, acting with objects) and pretend play (the child pretends to be something or someone, but does not create a plot with other players; Kravtsov and Kravtsova, 2017).

According to Vygotsky, role-play is a culturally determined phenomenon including three key characteristics (1) an imaginary situation, (2) roles and role actions, and (3) a set of interrelated play rules. An imaginary situation distinguishes a child’s play activity from every other form of activity (Vygotsky, 2016). Imaginary situations include a plot, roles, attributes of the play, rules, and role-playing actions. Creation of an imaginary situation becomes possible on the basis of the separation of the visual field and the field of meanings—the ability that develops in the preschool age (Vygotsky, 2016). “In play, the child learns to act not in a visible, but in an intellective, i.e., in an imaginary situation, relying on internal tendencies and motives as opposed to motives and impulses deriving from the object” (Vygotsky, 2004, p. 66). While describing the “hospital play” of children, Vygotsky writes that “in the play the child cries like a patient, and at the same time rejoices like a player” (Vygotsky, 2004, p. 69). The child portrays a patient who faces a disease, but on the other hand, the child is not a patient—there is a discrepancy, a gap between the child and her role. “Action in a situation that is not seen, but only conceived mentally in an imaginary field (i.e., an imaginary situation), teaches the child to guide his behavior not only by immediate perception of objects or by the situation immediately affecting him but also by the meaning of this situation” (Vygotsky, 2016, p. 12). Collectively created imaginary situations represent unique, original, and exclusive relations of participants, shaped and framed by roles, rules, and collective actions which exist and develop only within the imaginary situations (Walker et al., 2020).

### Dramatic Perezhivanie and imaginary situation as a microsocial situation of development

Vygotsky mentioned a case of three children with the mother suffering from several nervous and psychological disorders. When drunk, the mother regularly beat the children (Vygotsky, 2019). Despite being in the same terrifying situation, the development of the three children became disrupted in different ways. Thus, the same circumstances resulted in an entirely different picture for the three children. Speaking about aspects (moments) of social environment Vygotsky highlights: “it is not in itself this moment or that moment, taken without regard to the child, but that moment, refracted through the perezhivanie of the child, which is able to define how that moment will affect the course of future development.” (Vygotsky, 2019, p. 70).

So we also can assume that the creation of an imaginary situation itself does not promote development within play. The situation should personally affect the child and be *lived through* by the child. The differentiation of three concepts: (1) social environment, (2) social situation, and (3) social situation of development (SSD) might be helpful when it comes to role-play and development (Veresov, 2019). The social environment is the wider context of life; the social situation is a concrete component of the wider social environment and the social situation of development (SSD) is “a completely original, exclusive single and unique relation between the child and reality” (Vygotsky, 1998, p. 198). In a single social situation several social situations of development can emerge. Being in the same social situation, different children demonstrate different developmental outcomes because the same situation is refracted by different perezhivanie (Veresov, 2019). The social environment is thus the source of development; it influences the child, but what turns the social situation into SSD is perezhivanie (Fleer et al., 2017).

Veresov (2019) suggests the theoretical development of the classic Vygotsky model “social environment - social situation - perezhivanie - SSD”—a theoretical dyad of dramatic perezhivanie and the microsocial situation of development. Dramatic perezhivanie is a special type of perezhivanie as a refraction of a dramatic collision that children experience. Introducing dramatic perezhivanie opens an opportunity to link on a theoretical level the concept of perezhivanie and the principle of development, as “… the basic principle of the functioning of higher functions … is social, entailing interaction of functions, in place of interaction between people. They can be most fully developed in the form of drama” (Vygotsky, 1989, p. 59). Dramatic perezhivanie refers to the contradictory nature of human development—there is no development without conflicts and dramas. Those are refracted through dramatic perezhivanie (Veresov, 2019). Dramatic perezhivanie contains the potential to become a turning point in a child’s development, it represents a short-term “microsocial situation of development” and corresponds to the main characteristics of the macrosocial situation of development associated with the age crisis (Veresov, 2019).

An imaginary role-play situation could be seen as a specific microsocial situation. The general sociocultural context that determines the content of the play, surrounds children, and exists independently of them, represents the social environment. In play, all the children belong to a common play situation, which may or may not affect their development. Every complex sociodramatic role-play includes numerous micro-crises and contradictions (an argument with playmates, the need to follow the rules or the plot, to wait one’s turn, to play a role that was given by other players, etc.). The child might experience internal micro-dramas in play when she has to constantly restrain her impulsive reactions in order to remain within the imaginary situation in play. Thus, as a social situation of a special kind, play potentially includes a series of microsocial situations of development of EFs.

The following sections of the paper present experimental findings on the influence of a role-play intervention on young children’s EF development. The data are analyzed through the concepts of dramatic perezhivanie, micro-crisis, and microsocial situation of development. It will be argued that the dramatic perezhivanie might be considered a driver of EFs’ development through role-play in early childhood.

## Experimental study

### Aims of the study

The research presented in this paper aimed to empirically examine the dramatic perezhivanie as a factor mediating the effect of play intervention on child’s EFs development. We set the following goals:

1. to identify behavioral patterns of dramatic perezhivanie in a role-play and explore the very possibility of registering it in a role-play;
2. to describe individual trajectories of EFs’ development by comparing observation data and the results of the EFs’ assessment;
3. to identify if different types of adult engagement in a role-play influence dramatic perezhivanie patterns and results of play intervention regarding EFs development.

### Design

The experiment was conducted in 2020 on a Russian sample of preschoolers (n = 199, 46.7% females, 4.5–5.5 years old). Randomized experimental design with repeated measures was used. The study consisted of a preliminary assessment of executive functions levels (September–October); a 7 week-long intervention (October–November); and the testing of executive functions immediately after the intervention (December). The children were divided into groups equal in gender, age, and the initial level of executive function development. In all the experimental groups, the children played in different ways for 20–30 min twice a week: three types of role-play (free role-play; adult-directed play; child-directed play), digital games, and games with rules (board games). In the control group, the play was replaced by drawing on the theme of the story being read by the experimenter.

The testing of executive functions was based on the Miyake model (Miyake et al., 2000). Five tests were used. The NEPSY-II (Korkman et al., 2007) subtests were used to assess the level of development of verbal working memory (Sentences Repetition, SR), visual working memory (Memory for Designs, MD) and cognitive inhibition control (Inhibition). As its authors state, NEPSY-II is based on Luria’s ideas and subtests are similar with tests developed in line with cultural-historical psychology and activity theory. The Dimensional Change Card Sort (DCCS) test was used to assess switching (Zelazo, 2006). Assessment of the motor persistence was conducted using the “Statue” test. The child’s task was to maintain a motionless position with eyes closed for 75 s, restraining impulsive reactions in response to distracting sounds. The researchers were fully aware of the limitations of these assessment tests—their quantitative nature and static nature. However, for research purposes (pre and post testing) and for research questions, they seem to be applicable and reliable.

### Data collection

The assessment was conducted individually in a quiet room. For two experimental conditions (adult-directed play; child-directed play), video recording was organized. The videos were only available to the researchers, so the confidentiality of the children involved was maintained. The Ethics Committee approved the study and consent procedures of the Faculty of Psychology at Lomonosov Moscow State University (approval no: 2021/72). The parents of each participant gave written consent for their child to participate in the study and their permission to videotape the child.

In both videotaped experimental conditions, the children took on the same roles, made costumes, and acted out the story. In “Adult-directed play” intervention the adult controlled the distribution of roles and the plot development. The adult told the story, and the children were supposed to act in consistency with the plot and their roles. This condition reduced the load from the working memory, as the key aspects of the play were handled by the adult. The adult also reminded the child of his/her role if necessary (addressed him/her using the character’s name, reminded him/her of the role features: ‘you are a great magician’, etc.) and assisted children in playing the role (‘let us do it like if you were…’). In other words, the adult accentuated and supported the functions of inhibition and shifting. He/she also basically set for the children an example of ‘mature’ role-play and taught them how to play. For this experimental condition, scenarios were composed for each session, one story per gathering.

In “Child-directed play” intervention, the adult helped one child to take the director’s position: to distribute the roles, to make up a story, and play it with other children. Thus, there was no scenario prepared and an adult was there only to help when needed. The load on a directing child’s working memory was higher (as he/she controlled the plot and had to remember the roles of all participants). The directing child also had to apply higher inhibition (to follow the role of the director-in-charge and regulate other children’s behavior) and shifting (this child guided the general plotline and the actions of all the characters). Meanwhile, for other participants, the invoked developmental mechanisms were identical to the ones in the “Adult-directed play” option. One play session included two stories with two children performing the director’s functions. All participants had equal opportunities to take this position, and on average each had to do it 4 times.

Two digital cameras were used simultaneously in order to increase the coverage. In total over 70 h of video, material was obtained in the study. Given the overall amount of material, only the first and last play sessions were chosen for the analysis. Twenty children (10 from each group) participating in the “Child-directed play” and the “Adult-directed play” were selected for the analysis of the videos. The selection criteria included the attendance of at least 8 sessions, as well as the availability of results of the “The Dimensional Change Card Sort” (DСCS) and “Inhibition” tests (children with high, average, and low scores on these tests were selected into the sample).

### Data analysis

For data analysis, we applied a tool built within the framework of cultural-historical tradition. The Play Matrix is a tool for structured observation and includes a number of behavioral indicators combined into three blocks: actions, emotional manifestations, and speech behavior (Veraksa et al., 2022a, b). The category of actions includes such behavioral indicators as role actions, original role actions, field and impulsive actions; voluntary actions outside the play context; and actions in different play contexts. The category of emotional manifestations covers expressive movements and emotional reactions, emotional shouts, and emotionally saturated group actions. The category of speech behavior includes role statements, suggestions on how to act next, regulation of other children’s behavior, comments, play-related judgments, and meta-reflexive utterances. The Play Matrix allows evaluation of play not based on the external criteria of the observer, but as a situation experienced by a particular child. We can see how a child is engaged in play, if she seeks to enact the characters as expressively as possible or acts rather formally.

For our analysis, we focused on several indicators of the regulatory and emotional components of a child’s behavior:

1. impulsive actions;
2. field actions (actions that are defined by aspects of the field, environment – for example, a child while playing sees picture on the wall and begins to talk about it despite of ongoing play and one’s role in it);
3. regulation of other children’s behavior (a child tells other children what and how they should/should not do);
4. original role actions (for example, a child thinks up atypical actions or attributes for his or her character);
5. expressive movements and emotional response reactions (smiles, gestures, etc.).

The indicators No 3–5 were chosen as they represent the engagement of a child into play: if a child is emotionally engaged, if she seeks to influence the general plot and takes her role seriously. The indicators No 1 and 2 in turn were chosen as the indicators of a child’s self-regulation within the play; they capture the moments when a child “drops out” of the plot or cannot relate her character actions with the general plot. Analysis of these behavioral manifestations as well as their combination within each microsocial situation allowed us to catch the moments of contradiction and dramatic perezhivanie, which can become a turning point in a child’s development.

At the first stage of the analysis, detailed protocols of the playing sessions were created which registered every behavioral manifestation of the child during the session. The next step was to attribute each of the child’s manifestations to one or several of the indicators. The number of fixations of each behavioral indicator was counted and entered into the summary table (see Appendixes 1a,b).

The statistical analysis for the two experimental groups was supplemented with additional data including observations. Individual differences in the effect of the play intervention were examined by comparing the observations and test results of individual children.

## Results

### Behavioral patterns of dramatic perezhivanie in a role-play

The analysis of the video recordings revealed the following general tendencies.

In the “Child-directed play” group, some children derive special pleasure from, and devote much attention to, the regulation of other children’s behavior, even if they are not directors. They actively indicate and show other children how to do this or that role action. They express displeasure at not being listened to or doing things “the wrong way.” While performing their roles they keep interfering with other children’s role-play or the director’s story. They disagree and reject the director’s story moves if they do not like them. In the “Adult-directed play” group, some children also tend to regulate other children’s behaviors when it greatly interests them, so eager to play that they begin to help other children play their roles and direct them.

Impulsive and field actions are clearly evident in several situations. If a child is emotionally involved in play, one performs impulsive actions out of impatience and due to overwhelming emotions. If there is a pause in play and/or the child becomes bored, then impulsive as well as field actions become a way to entertain oneself. This includes a situation when a child or several children continue to play “their game” in spite of the director’s instructions. For example, they start to fight again, even though the battle is already over according to the plot. In this case, the observer records a considerable number of impulsive and original actions – the child does possess his or her own creativity in acting out the role, but it does not fit into the general storyline and the other characters’ actions and runs parallel.

At the same time, there are children who do not manifest impulsive or field actions even if their character is underrepresented in the story. They wait calmly and observe the play attentively. This can be inferred from their emotional reactions to what is going on—it is a quiet smile often. However, these children also perform their roles in a stiff and stereotypical way. For example, a boy, playing the part of a dragon, simply strolls to where the dragon is supposed to be flying according to the storyline. And even at the last sessions, he has a hard time with his lines, only when the adult gives him the cue, does the child very quietly utter the offered line.

Thus, we can see that the role-play contains multiple situations of contradictions and micro-crisis which can trigger dramatic perezhivanie.

### Individual trajectories of EFs development

As mentioned above, play is full of contradictions by virtue of its nature and can serve as a source of dramatic perezhivanie for a playing child. However, there can be different ways of resolving the contradiction that underlies dramatic perezhivanie. Let us illustrate it with some examples.

The girl ‘L.’ (in the “Adult-directed play” condition) displays a great number of bright emotional reactions and expressive movements. For example, she interacts with the adult easily and swiftly, asking questions about the play; coming up with an attribute for her character and proudly declaring: “This is a sword!”; waiting for her turn to move onstage in the story and listening attentively; and getting scared of the cave monster in an expressive and serious way. L. wants to act, but she has to wait her turn and consider other characters and the plot. There is a struggle of motives—dramatic perezhivanie, a micro-crisis—the resolution of which requires switching on self-regulation skills.

L.’s pre-test results are in the average range, and this situation could well become developmental. However, we can see that “I.” often jumps up and down with impatience, and runs around the room, eager to play even if the plot has nothing to do with her character at this particular moment. In essence, self-regulation mechanisms do not work under conditions of intense affect. According to L.’s post-test results, positive dynamics is observable only in respect of visual working memory. There were no positive dynamics in indicators of cognitive flexibility, cognitive inhibitory control, and verbal working memory. A slight decrease in the indicators was registered in relation to physical inhibitory control, which was manifested in playing sessions.

Another example of resolving a similar micro-crisis is the boy “S.” (in the “Child-directed play” condition). His pre-test results are also in the average range. The child also shows manifestations of great involvement, both at the beginning and at the end of the experimental series. For example, he shows initiative and desire to be the director at the very first session when the situation is still new to the children. When he fails to get the role of the Martian in the last session, he is greatly annoyed and says “aaah” but when he does get the coveted role, he becomes overwhelmed. When the plot is stalling (the director takes too long to think up the next move), the boy asks the adult “well?” anxious to see what happens next. When the boy S. performs his role actions, he does them as best as he can: he prostrates himself on the floor pretending to be fast asleep according to the plot. Very much like the girl L., S. displays manifestations of impulsive behavior, but it happens less often, and the episodes are overall shorter – the boy returns to the game by himself and continues to follow the story and his role. On one occasion when S. fails to follow the director’s instructions, the experimenter draws his attention. S. explains that he “did not hear it” and continues to follow what is going on very attentively. The experimenter’s involvement had a much shorter and weaker effect on the girl L. According to the post-test results of the boy S., we observe positive dynamics in the level of development of cognitive inhibitory control and visual working memory and a small increase in indicators of cognitive flexibility.

One more example of the child’s interaction with the play situation is shown by boy ‘L.’ (“Child-directed play”). As intervention goes by, the boy L. loses his interest in the playing sessions quite quickly, so the play situation does not cause any dramatic perezhivanie. This leads to a significant increase in impulsive actions by the end of the experiment. For example, after hearing a girl mention Basilio the cat, he interrupts and shouts “hey, Basilio the cat!” to no one in particular. The boy often inserts cues or makes actions outside of the plot development, which amuses and distracts other children. He talks spontaneously to a friend and gets distracted from what is going on in play. He peeks out into the hallway to see the goings-on there. The experimenter often needs to invite the boy L. into the play and repeat the story about his character twice to get him “back.” It is interesting that the boy L.’s pre-test results are in the high range; however, the play situation which fails to involve the child on a personal level does not lead to inclusion and development of his self-regulation skills. According to the post-test results, there is no positive dynamics in scores of inhibition, physical inhibitory control, and visual working memory; there was even a small decrease in scores of cognitive flexibility and verbal working memory.

The case analysis reveals a role-play as a microsocial situation which is not the social situation of development *per se*, but can become such for each specific child at the moment when dramatic perezhivanie occurs.

### The influence of the type of adult engagement

Detailed results of statistical analysis are presented by Veraksa et al. (2022a, b), Veresov et al. (2021). *T*-test was applied for the comparison of the pre- and post-test results for each executive functions’ measure. All the variables in the analysis were normally distributed (*p* > 0.05 according to the Kolmogorov–Smirnov test). In the ‘Adult-directed play’ intervention group (*N* = 29; age in months 60, 30) significant changes were registered in 4 (out of 5) measures of executive functions: inhibition control, motor persistence, shifting, visuospatial working memory. Verbal working memory was the only measure that did not show significant positive dynamic under this condition. However, this intervention was designed to take the load from this memory type due to the presence of an adult director. In the “Child-directed play” (*N* = 38; age in months 61, 21) significant changes were found in three measures of executive functions: in all except verbal working memory and motor persistence (Table 1).

**Table 1:** Pre- and post-test differences in measures of executive functions within two experimental conditions of interest: “Child-directed play” and “Adult-directed play.”

Executive Functions measures		Pre-test		Post-test		*p*
		*M*	SD	*M*	SD
Adult-directed play	Cognitive flexibility^*^	17.71	2.59	19.15	2.67	*t*(26) = −2.84 *p* = 0.009
	Verbal working memory	16.32	3.95	17.18	4.14	*t*(26) = −1.31, *p* = 0.201
	Visual working memory^*^	63.54	14.88	72.79	20.14	*t*(22) = −2.48, *p* = 0.021
	Motor persistence^*^	22.96	6.30	27.29	5.10	*t*(22) = −3.20, *p* = 0.004
	Inhibition control^*^	9.75	3.03	11.92	3.26	*t*(26) = −3.02, *p* = 0.006
Child-directed play	Cognitive flexibility^*^	17.92	1.93	19.24	2.55	*t*(37) = −2.98, *p* = 0.005
	Verbal working memory	18.31	3.11	18.68	2.91	
	Visual working memory^*^	60.78	16.16	71.11	19.83	*t*(36) = −3.51, *p* = 0.002
	Motor persistence	27.39	2.99	28.00	3.04	
	Inhibition control^*^	8.21	3.38	11.92	3.26	*t*(36) = −6.31, *p* < 0.001

*significant changes were registered.

To explore further, we used observation according to chosen behavioral indicators to identify the features of the microsocial situation set by each type of role-play. It seems rather clear that when the adult directs the play, the control is stronger and the responsibility for the storyline rests on the adult rather than on a child. But when the child does the directing, one can express oneself to a greater extent. We surmised that such play should be more emotionally meaningful for the child. Statistical analysis methods were applied to process the data obtained from observation. As we analyzed the initial sessions and those at the end of the intervention, we had the opportunity to analyze the deltas (differences between the last and first play sessions).

Due to the small sample size, the nonparametric Mann–Whitney test was used. It was found that the number of expressive movements and emotional reactions at the end of the intervention was significantly higher (*U* = 22.000, *p* = 0.032) in the group of children where they themselves were directors than in the group of children where the director was an adult. The difference in the number of original role actions is significantly lower (*U* = 25.500, *p* = 0.062) at the beginning and at the end of the intervention at the tendency level, and expressive movements and emotional reactions are significantly higher (*U* = 27.500, *p* = 0.086) in the group of children where they directed themselves than in the group of children where an adult was the director. The latter means that at the tendency level, the number of original role actions increased as a result of the “Adult-directed play” intervention. In addition, in the group where children were directors, the number of expressive movements and emotional reactions increased. Indeed, role performance and storyline in the “Child-directed play” condition remained rather stereotypical even at the end of the intervention. Children tend to include fights (battles), death and resurrection, weddings, rambles, transformations into a toad, sleep, and meals. The plot remains more coherent and consistent in the “Adult-directed play” group. This seems to create a condition for original manifestation in children. The adult’s story can be relied upon to come up with something of one’s own. In the “Adult-directed play” condition, the story has more character interactions and general logic and various plot twists. At the same time, the experimenter’s “dictatorship” in the play leads to a situation where the children’s manifestations are generally poorer, as can be seen in the summary table (see Appendix). However, even the most reluctant and shy children start to resist the imposed plot towards the final sessions, inventing and acting out something of their own, at least in trifles. The final sessions show that the play often gets out of the adult’s control, and the children’s plot runs parallel to that of the adult’s. This confirms the idea that a certain degree of freedom in play is necessary to make it meaningful, more emotional, and “lived through” for children.

## Discussion

Vygotsky differentiated two ways that perezhivanie can be conceptualized: as an empirical phenomenon available for observation and assessment (P1) and as a theoretical tool for examination of the development (P2; Veresov, 2016). In our work, we were challenged to explore the power of the theoretical tool in regard to the role-play and to operationalize empirical phenomena to make it really available for observation and assessment in the role-play. Through the concepts of “microsocial situation of development” and “dramatic perezhivanie” an imaginary situation, which is created within the role-play is analyzed as a specific microsocial situation turning into microsocial situation of development for a particular child in the moment of dramatic perezhivanie. We focused on impulsive, field actions, regulation of other children’s behavior, original role actions and expressive movements, and emotional response reactions. We assumed that these behavioral manifestations as well as their combination within each microsocial situation allow us to catch the moments of contradiction and dramatic perezhivanie, which can become a turning point in a child’s development.

The case analysis shows that we can consider a role-play as a microsocial situation which is not the social situation of development *per se*, but can become such for each specific child at the moment when dramatic perezhivanie occurs. Dramatic perezhivanie can indicate the degree of the child’s involvement in what is happening, and the degree of this involvement allows us to characterize a play situation as a social situation of development. Thus, the behavioral indicators that capture the child’s actions, emotional manifestations and speech behavior, can become a tool enabling us to analyze the developmental potential of play. In a broader sense, analysis of dramatic perezhivanie can show which parts, aspects, or components of a situation are reflected in the child’s mind, what he responds to and to what he does not. Following Vygotsky (2016), we can say that in play the child is always “a head above himself.” However, our data have shown that sometimes this may not be the case. If the play is rich in content, if the child is active and involved in it, we can say that the play situation has a developing potential. But the same play situation may be experienced differently by another child. If the play is not interesting and is weak in content for the child, then it does not contribute to development, and does not show the actual level of the child’s development. We saw from the case study that a child in play can be “a head lower himself” comparing the test results and play behavior. This result seems to show how “hot” self-regulation interferes and connects to the “cold” self-regulatory processes in a way the problem of the unity of affect and intelligence is stated in cultural-historical theory (Veraksa and Sukhikh, 2021). Сold (cool executive control, CEC) and hot control (hot executive control, HEC) are two important and interrelated aspects of self–regulation. CEC is more effectively neutral, slow-acting, and developing; HEC is more reflex, fast-acting, early-developing, and stimulus-dependent (Willoughby et al., 2011). Unlike CEC, HEC is launched in the context of emotional and motivational processes. The role-play is the most natural context for the manifestation of such processes in preschoolers. Probably, the Play Matrix can also be the tool for assessing HEC in a personally significant situation, which is (often, but not always, as we see) a joint role-play.

The data analysis also provide some grounds to support the suggestion that different conditions of play vary in developmental potential. We observed different trends in children’s behavior depending on the experimental condition. Specifically, children seem to be more involved and active in the context of peer play (“Child-directed play”). At the same time, at the tendency level, the number of original role actions increased as a result of the “Adult-directed play” intervention. This experimental condition also became more efficient for behavioral inhibition development. Compared to less guided forms of role-play, articulated and active participation of adults contributes to children’s ability to suppress impulses and automated reactions. This finding points to Vygotsky’s perspective regarding the role of an adult in play: an adult is important during play but her involvement should be limited. As previous studies also showed, the adult has to keep a balance when engaging in children’s play (Hakkarainen et al., 2013; Fleer, 2015; Smirnova, 2019). The adult is indispensable for play development as a carrier of cultural norms and experience; on the other hand, support for children’s initiative is equally necessary for play to be personally meaningful and emotionally engaging for the child.

The study shows that children’s behavioral, emotional and speech manifestations can be seen as indicators of a child’s attitude toward play and can be used to describe the phenomenology of dramatic perezhivanie. Obvious limitations of this study are its small sample size and newly developed observational tool (not used in prior work). An original tool, *The Play Matrix* has an extensive number of indicators and requires training for observer. So we had to choose only five behavioral indicators. We believe it would be important to continue working on the development, refinement, and validation of a system of behavioral indicators that allow to analyze play from the point of view of a particular child and more accurately capture the moments of dramatic perezhivanie in play. There are very limited reports on the perezhivanie that are operationalized within empirical research (Fleer, 2016; Ferholt, 2018; Veresov, 2019) and there are no tools for dramatic perezhivanie to be observed and captured in a role-play. However, such tools, especially available for use by educational practitioners, would make it possible to predict and increase the effectiveness of interventions for each individual child, which is consistent with the idea of individualization of the educational process. Another possible direction for further research could be to study the effectiveness of play interventions taking into account children’s dramatic perezhivanie and their personality traits. Finally, it could be fruitful to further apply the suggested methodology to analyze the results of interventions aimed to develop not only executive functions but other cognitive skills and lines of development as well.

## Conclusion

Adopting a framework of the cultural-historical approach, we explore the phenomena of dramatic perezhivanie as a driving force for children’s development. We used the data from an experimental study aimed at developing children’s executive functions. The play activities in this experiment were specifically designed to provide a load on these cognitive skills. However, the methodological principle proposed in this article for the qualitative analysis of the developmental effect of intervention can be applied to any material. We can create special conditions for the development of certain skills, but the created environment itself does not develop and a microsocial situation will not become a turning point in the child’s development if it is not possible to involve him personally and create a microsocial situation of development through dramatic perezhivanie. We assume that this is the general mechanism of development in role-play, and in any other (micro) social situation.

## Data availability statement

The original contributions presented in the study are included in the article/Supplementary material, further inquiries can be directed to the corresponding author.

## Ethics statement

The studies and consent procedures involving human participants were reviewed and approved by the Ethics Committee of Faculty of Psychology at Lomonosov Moscow State University (the approval no: 2021/72). Written informed consent to participate in this study was provided by the participants’ legal guardian/next of kin. Written informed consent was not obtained from the individual(s) for the publication of any potentially identifiable images or data included in this article.

## Author contributions

VS obtained data, contributed to methods analyses and preparation of the manuscript. NNV conceptualized research and contributed to results interpretation. NEV introduced theoretical analyses and co-directed the analyses. All authors contributed to the article and approved the submitted version.

## Conflict of interest

The authors declare that the research was conducted in the absence of any commercial or financial relationships that could be construed as a potential conflict of interest.

## Publisher’s note

All claims expressed in this article are solely those of the authors and do not necessarily represent those of their affiliated organizations, or those of the publisher, the editors and the reviewers. Any product that may be evaluated in this article, or claim that may be made by its manufacturer, is not guaranteed or endorsed by the publisher.

## Supplementary material

The Supplementary material for this article can be found online at: https://www.frontiersin.org/articles/10.3389/fpsyg.2022.1057209/full#supplementary-material
