# Supplementary material for: Dramatic Perezhivanie as a driver of executive functions development through role-play in early childhood: Theoretical framework and experimental evidence
Source: Front Psychol. 2022 Nov 18;13:1057209. doi: 10.3389/fpsyg.2022.1057209 (PMC9743294; doi:10.3389/fpsyg.2022.1057209)
Supplement: Supplementary file 1 [file Data_Sheet_1.docx]

**Appendix 1a.** The Adult-directed play: results of video processing using the chosen behavioral indicators.

The first line for each child means the first session, the second line is the last one. The results of the executive functions’ (EF) tests are presented in a similar way: pre-test (first line) and post-test (second line).

| Children | Actions | | | Emotions | Speech | EF testing | | | | | |
| --- | --- | --- | --- | --- | --- | --- | --- | --- | --- | --- | --- |
|  | Impulsive | Field | Original | Expressive movements and emotional reactions | Regulation of other children’s behaviors | DCCS | SR | Inhibition (naming and inhibition) | | MD | Statue |
| L. [girl] | 8 | 1 | 1 | 11 | 1 | 19 | 16 | 10 | 8 | 89 | 17 |
|  | 5 | 3 | 10 | 5 | 0 | 18 | 13 | 7 | 8 | 98 | 14 |
| I. [boy] | 3 | 0 | 1 | 5 | 3 | 17 | 11 | 10 | 7 | 80 | 21 |
|  | 0 | 0 | 0 | 1 | 1 | 18 | 14 | 10 | 7 | 80 | 29 |
| N. [girl] | 0 | 0 | 1 | 6 | 1 | 19 | 20 | 10 | 10 | 69 | 23 |
|  | 0 | 0 | 0 | 3 | 0 | 16 | 19 | 14 | 11 | 81 | 30 |
| V. [boy] | 3 | 4 | 5 | 4 | 1 | 24 | 19 | 14 | 13 | 75 | 28 |
|  | 2 | 1 | 9 | 2 | 1 | 24 | 19 | 15 | 18 | 67 | 29 |
| D. [boy] | 5 | 4 | 0 | 6 | 0 | 16 | 14 | 4 | 5 | 53 | 29 |
|  | 22 | 0 | 13 | 3 | 1 | 17 | 16 | 14 | 13 | 94 | 22 |
| G. [boy] | 0 | 0 | 2 | 3 | 0 | 17 | 13 | 7 | 8 | 64 | 24 |
|  | 2 | 2 | 9 | 1 | 1 | 18 | 14 | 11 | 8 | 65 | 30 |
| К. [girl] | 13 | 1 | 8 | 2 | 2 | 22 | 25 | 14 | 12 | 56 | 13 |
|  | 0 | 1 | 10 | 0 | 8 | 22 | 20 | 14 | 14 | 72 | 29 |
| К. [boy] | 0 | 0 | 0 | 2 | 0 | 19 | 15 | 10 | 8 | 61 | 30 |
|  | 1 | 3 | 0 | 0 | 2 | 20 | 17 | 14 | 10 | 84 | 29 |
| М. [girl] | 0 | 3 | 2 | 0 | 0 | 19 | 14 | 8 | 12 | 74 | 29 |
|  | 0 | 3 | 8 | 1 | 2 | н | 10 | 14 | 13 | 79 | 30 |
| М.^1^ [boy] | 2 | 0 | 0 | 3 | 1 | 16 | 11 | 4 | 6 | 49 | 21 |
|  | 17 | 6 | 14 | 2 | 7 | 15 | 17 | 5 | 3 | 53 | 25 |

**Appendix 1b.**

The Child-directed play: results of video processing using the chosen behavioral indicators. The first line for each child means the first session, the second line is the last one. The results of the executive functions’ (EF) tests are presented in a similar way: pre-test (first line) and post-test (second line).

* - the child acted as director in this session.

| Children | Actions | | | Emotions | Speech | EF level testing | | | | | |
| --- | --- | --- | --- | --- | --- | --- | --- | --- | --- | --- | --- |
|  | Impulsive | Field | Original | Expressive movements and emotional reactions | Regulation of other children’s behavior | DCCS | SR | Inhibition (naming and inhibition) | | MD | Statue |
| А. [girl] | 0* | 0* | 1* | 11* | 19* | 18 | 16 | 3 | 6 | 75 | 27 |
|  | 0* | 0* | 3* | 2* | 12* | 24 | 17 | 13 | 12 | 69 | 28 |
| D. [boy] | 3* | 0* | 1* | 1* | 2* | 17 | 21 | 10 | 10 | 57 | 27 |
|  | 1* | 1* | 1* | 3* | 2* | 20 | 22 | 14 | 14 | 93 | 28 |
| S. [boy] | 2* | 2* | 3* | 7* | 6* | 19 | 23 | 5 | 9 | 87 | 30 |
|  | 5 | 8 | 3 | 5 | 1 | 21 | 22 | 15 | 15 | 111 | 30 |
| А. [girl] | 17 | 0 | 1 | 11 | 1 | 17 | 19 | 11 | 9 | 63 | 28 |
|  | 19* | 2* | 3* | 1* | 7* | 19 | 18 | 10 | 14 | 71 | 29 |
| V. [boy] | 2 | 3 | 1 | 6 | 1 | 20 | 21 | 12 | 14 | - | - |
|  | 5 | 0 | 2 | 14 | 1 | 22 | 18 | 17 | 17 | 67 | 28 |
| L. [boy] | 2* | 1* | 6* | 1* | 0* | 23 | 25 | 13 | 10 | 56 | 28 |
|  | 18 | 7 | 9 | 1 | 3 | 19 | 20 | 14 | 10 | 58 | 28 |
| E. [girl] | 11* | 0* | 1* | 5* | 8* | 16 | 15 | 4 | 5 | 65 | 28 |
|  | 5 | 1 | 2 | 6 | 0 | 17 | 20 | 10 | 9 | 51 | 29 |
| А. [girl] | 0 | 0 | 4 | 2 | 1 | 17 | 15 | 7 | 9 | 73 | 29 |
|  | 2 | 3 | 4 | 3 | 2 | 20 | 15 | 12 | 15 | - | - |
| S.^*^ [boy] | 3* | 3* | 7* | 3* | 25* | 17 | 18 | 6 | 5 | - | - |
|  | 6* | 5* | 6* | 8* | 25* | 18 | 18 | 8 | 9 | 57 | 30 |
| М. [girl] | 11 | 0 | 7 | 6 | 4 | 18 | 19 | 14 | 14 | 55 | 28 |
|  | 4 | 1 | 2 | 5 | 5 | 16 | 19 | 15 | 11 | - | - |
